# An Open-label, Randomized Study of Melphalan/Hepatic Delivery System Versus Best Alternative Care in Patients with Unresectable Metastatic Uveal Melanoma

**DOI:** 10.1245/s10434-025-17231-x

**Published:** 2025-04-07

**Authors:** Jonathan S. Zager, Marlana Orloff, Pier Francesco Ferrucci, Junsung Choi, David J. Eschelman, Evan S. Glazer, Aslam Ejaz, J. Harrison Howard, Erika Richtig, Sebastian Ochsenreither, Sunil A. Reddy, Michael C. Lowe, Georgia M. Beasley, Anja Gesierich, Armin Bender, Martin Gschnell, Reinhard Dummer, Michel Rivoire, Ana Arance, Stephen William Fenwick, Joseph J. Sacco, Sebastian Haferkamp, Carsten Weishaupt, Johnny John, Matthew Wheater, Christian H. Ottensmeier

**Affiliations:** 1 Department of Cutaneous Oncology, Moffitt Cancer Center, Tampa, FL USA; 2 Department of Oncologic Sciences, University of South Florida, Morsani School of Medicine, Tampa, FL USA; 3 Thomas Jefferson University, Philadelphia, PA USA; 4 European Institute of Oncology, IRCCS, Milan, Italy; 5 Moffitt Cancer Center, Tampa, FL USA; 6 The University of Tennessee Health Science Center, Memphis, TN USA; 7 The Ohio State University, Columbus, OH USA; 8 University of South Alabama, Mobile, AL USA; 9 Medical University of Graz, Graz, Austria; 10 Charité Comprehensive Cancer Center, Berlin, Germany; 11 Stanford University, Stanford, CA USA; 12 Emory University, Atlanta, GA USA; 13 Duke University, Durham, NC USA; 14 University Hospital Würzburg, Würzburg, Germany; 15 University Hospital Marburg, Marburg, Germany; 16 University Hospital Zürich, Zürich, Switzerland; 17 Léon Bérard Center, Lyon, France; 18 Hospital Clínic Barcelona, Barcelona, Spain; 19 Liverpool University Hospitals, NHS Foundation Trust, Liverpool, UK; 20 The Clatterbridge Cancer Center, University of Liverpool, Liverpool, UK; 21 University Hospital Regensburg, Regensburg, Germany; 22 University Hospital Münster, Münster, Germany; 23 Delcath Systems, Inc., Queensbury, NY USA; 24 University Hospital Southampton, NHS Foundation Trust, Southampton, UK

**Keywords:** Metastatic uveal melanoma, Percutaneous hepatic perfusion, Melphalan/Hepatic Delivery System, Liver-directed therapy, Ocular melanoma

## Abstract

**Background:**

Metastatic uveal melanoma (mUM) has a poor prognosis, with liver metastases typically presenting a therapeutic challenge. Melphalan/Hepatic Delivery System (Melphalan/HDS) is a drug/medical device combination used for liver-directed treatment of unresectable mUM patients. This study assessed efficacy and safety of Melphalan/HDS versus best alternative care (BAC).

**Methods:**

Eligible patients with unresectable mUM were randomized (1:1) to receive Melphalan/HDS (3 mg/kg ideal body weight) once every 6 to 8 weeks for a maximum of 6 cycles or BAC. Due to slow enrollment and patient reluctance to receive BAC treatment, the study design was amended to a single-arm Melphalan/HDS study, and all efficacy analyses of the randomized study were treated as exploratory.

**Results:**

The study enrolled 85 patients. Eligible patients were randomized to receive Melphalan/HDS (*n* = 43) or BAC (*n* = 42), and 72 patients received study treatment (Melphalan/HDS [*n* = 40]; BAC [*n* = 32]). Exploratory analyses of efficacy endpoints showed numerical differences consistently favoring the Melphalan/HDS arm versus BAC (median overall survival: 18.5 vs. 14.5 months; median progression-free survival: 9.1 vs. 3.3 months; objective response rate: 27.5% vs. 9.4%; and disease control rate: 80.0% vs. 46.9%). Serious adverse events (SAEs) occurred in 51.2% of Melphalan/HDS and in 21.9% of BAC patients. The most common (>5%) SAEs included thrombocytopenia (19.5%), neutropenia (9.8%), leukopenia (9.8%) and febrile neutropenia (7.3%) in Melphalan/HDS patients and cholecystitis, nausea and vomiting (6.3% each) in BAC patients. No treatment-related deaths were observed.

**Conclusion:**

Treatment with Melphalan/HDS shows clinically meaningful efficacy and demonstrates a favorable benefit-risk profile in patients with unresectable mUM as compared to BAC.

**Supplementary Information:**

The online version contains supplementary material available at 10.1245/s10434-025-17231-x.

Uveal melanoma (UM) is a rare malignancy accounting for approximately 3% to 5% of all melanoma cases and represents the most common intraocular malignancy in adults.^1^ Despite optimal management of primary tumors, up to 50% of patients with UM will develop metastatic disease (mUM), mostly to the liver (approximately 90% of cases). Once patients present with metastatic disease, their prognosis is poor, with typical median overall survival (mOS) of approximately 1 year.^1–3^

Contemporary regimens for systemic treatment of mUM include the novel bispecific gp100 peptide-HLA-directed CD3 T cell engager tebentafusp (which is limited to patients who are positive for HLA-A*02:01) and immune checkpoint inhibitors (ICI), such as pembrolizumab, nivolumab and ipilimumab. Tebentafusp has demonstrated a clinically meaningful improvement in OS, whereas ICI therapies rarely produce durable responses or significant survival benefit.^4,5^ Single agent or dual ICI regimens have shown limited efficacy in mUM patients with objective response rates (ORR) ranging from 0 to 16.7% in retrospective chart analyses.^6–9^ In prospective Phase 2 studies in mUM patients, the combination of nivolumab and ipilimumab showed ORR of 18% in a small, single-center study,^10^ and of 11.5% in a multicenter study.^11^ A recent systematic review and meta-analysis^12^ showed mOS, median progression free survival (mPFS) and overall ORR of 11.5 months, 3.0 months and 9.2% for ICI in mUM.

For the large majority of patients with mUM who have liver metastases, liver failure is a common outcome.^13^ Accordingly, liver-directed therapies are logical treatment options for this patient population. Current standards of care include liver-directed therapies such as transarterial chemoembolization (TACE), radioembolization or immunoembolization, thermal ablation as well as loco-regional perfusion procedures delivering high-dose chemotherapeutic agents: the surgical procedure intrahepatic perfusion (IHP) and the minimally invasive procedure percutaneous hepatic perfusion (PHP).^14,15^ PHP uses the Hepatic Delivery System (HDS), available in Europe as CHEMOSAT^®^ and in the US as HEPZATO KIT™ (Melphalan/HDS), which recently received approval by the US Food and Drug Administration (FDA).^16^

Liver-directed treatment should treat the whole liver and target all radiographically evident and occult metastases, allow for retreatment to optimize tumor control and minimize recurrence, and demonstrate an acceptable benefit/risk profile. TACE and radioembolization fulfill some of these requirements and generally achieve better control of hepatic metastases relative to systemic therapy. However, these procedures have limitations with respect to repeatability of treatment and ability to treat the whole liver. IHP, while demonstrating promising hepatic PFS and tumor response, as it perfuses the whole liver, is limited by the invasiveness of the procedure, which carries a high risk of morbidity and mortality; patients undergo only one treatment, which may significantly limit its use and patient outcomes.^17,18^ PHP is a minimally invasive technique optimized to saturate the whole liver with chemotherapy without the need for major surgery, and most patients are able to receive multiple treatments.

Findings from early-phase clinical studies investigating the safety and efficacy of PHP utilizing Melphalan/HDS in mUM patients show encouraging results, including promising ORRs and OS.^19–23^ The multicenter, open-label, Phase 3 FOCUS study was designed to evaluate the efficacy and safety of Melphalan/HDS in patients with unresectable mUM in comparison to best alternative care (BAC).

## Methods

### Patients

The study population included male or female patients 18 years of age or older who had histologically verified unresectable mUM to the liver, with up to 50% liver tumor involvement. Patients were previously treated or treatment-naïve, had Eastern Cooperative Oncology Group (ECOG)^24^ performance status of 0 to 1 at screening, had measurable liver metastases and could have limited extrahepatic disease that was amenable to resection or radiation.

Given that the PHP procedure requires general anesthesia and active coagulation/anti-coagulation control, the eligibility criteria were designed to minimize the risks associated with the procedure (e.g., exclusion of patients with moderate or severe liver cirrhosis, portal hypertension, New York Heart Association II-IV status). The eligibility criteria remained unchanged throughout the study. Detailed inclusion and exclusion criteria are described in the Supplementary Appendix.

### Study Design and Treatment

The study was initiated as a two-arm, open-label, controlled, randomized study conducted at 22 centers in the US and Europe. Eligible patients were randomized 1:1 to receive Melphalan/HDS or BAC (investigator’s choice of TACE, pembrolizumab, ipilimumab, or dacarbazine). Due to slow enrollment with patient reluctance to receive BAC treatment, the study design was amended to a single-arm study, after which all eligible patients received Melphalan/HDS.

Patients received melphalan (3 mg/kg ideal body weight; maximum dose of 220 mg for a single treatment) treatment once every 6 weeks for a maximum of six cycles. Prior to each treatment, liver venous outflow was isolated by a double-balloon catheter placed into the inferior vena cava. Melphalan was administered over 30 minutes via an infusion catheter placed in the hepatic artery. The infusion was followed by 30 minutes of washout with extracorporeal filtration to further reduce systemic exposure to melphalan. Patients received granulocyte-macrophage colony-stimulating factor (GM-CSF) within 72 hours of the PHP procedure to mitigate possible neutropenic effects of melphalan.

Treatment procedures were administered by a team of medical or surgical oncology, interventional radiology, anesthesiology, and a perfusionist. BAC treatments were administered in accordance with local prescribing information and institutional guidelines. Further details are included in the Supplementary Appendix.

### Endpoints and Assessments

The primary efficacy endpoint was overall survival (OS), and secondary efficacy endpoints were progression-free survival (PFS) and ORR, both as determined by investigators based on Response Evaluation Criteria in Solid Tumors (RECIST) version 1.1.^25^ Exploratory endpoints included duration of response (DOR), hepatic PFS and safety.

Adverse events (AEs) were assessed by investigators and graded according to National Cancer Institute Common Terminology Criteria for Adverse Events (CTCAE) version 4.03.^26^

### Study Oversight

The sponsor and all authors contributed to various elements of study design, protocol development, and data analysis. The protocol was approved by the institutional review board or independent ethics committee at each study center, as well as by all relevant competent authorities. This study was conducted in accordance with the principles of the Declaration of Helsinki and Good Clinical Practice guidelines, as outlined by the International Council for Harmonization. An independent data safety monitoring board provided oversight of safety. All participants provided written informed consent. All authors vouch for the accuracy and completeness of the data and for the fidelity of the study to the protocol.

### Statistical Analysis

The analysis plan was based on a planned sample size of approximately 240 patients (120 per treatment arm) and 192 OS events to provide 80% power to detect a difference between a mOS of 12 months in the Melphalan/HDS arm versus 8 months in the BAC arm (hazard ratio = 0.67) assuming an exponential distribution of OS event times and 0.05 significance level (two-sided). It was further assumed that study accrual would take 30 months with 12 months of follow-up. Final data analysis was to be performed when a total of 192 events occurred. One interim analysis of efficacy was planned for when approximately 50% of the total anticipated number of OS events (96/192 deaths) had occurred. Unless specified otherwise, statistical testing used a two-sided test at the 0.05 significance level.

However, because the randomized study was ended before enrollment of the full planned sample, all efficacy analyses performed on the randomized study were then considered exploratory, and nominal p-values are reported without control for study-wide type I error. Demographic data were summarized using descriptive statistics. Summary statistics for continuous variables included median and range (minimum/maximum). Categorical variables are presented as frequency counts and percentages. Time-to-event variables were summarized using Kaplan-Meier methods. For the calculation of time-to-event endpoints the start date was the randomization date. Descriptive statistics were used for safety analysis in the safety population, which included all patients for whom a study treatment or procedure was attempted. Analyses were performed using Statistical Analytics System (SAS) v9.4 or later.

## Results

### Patient Disposition and Baseline Characteristics

The planned study size was 240 patients; due to slow enrollment and patient reluctance to receive BAC treatment, the study design was amended to a single-arm Melphalan/HDS study, and all efficacy analyses of the randomized study were treated as exploratory.

From February 2016 to October 2018, 129 patients were screened. Of these patients, 85 met the eligibility criteria and were enrolled in the study. Treatment was attempted for 41 of the 43 patients randomized to the Melphalan/HDS arm, and 40 of these patients received treatment. The remaining patient did not receive treatment because of hepatic artery thrombosis noted before administration of study drug.

**Fig. 1:**
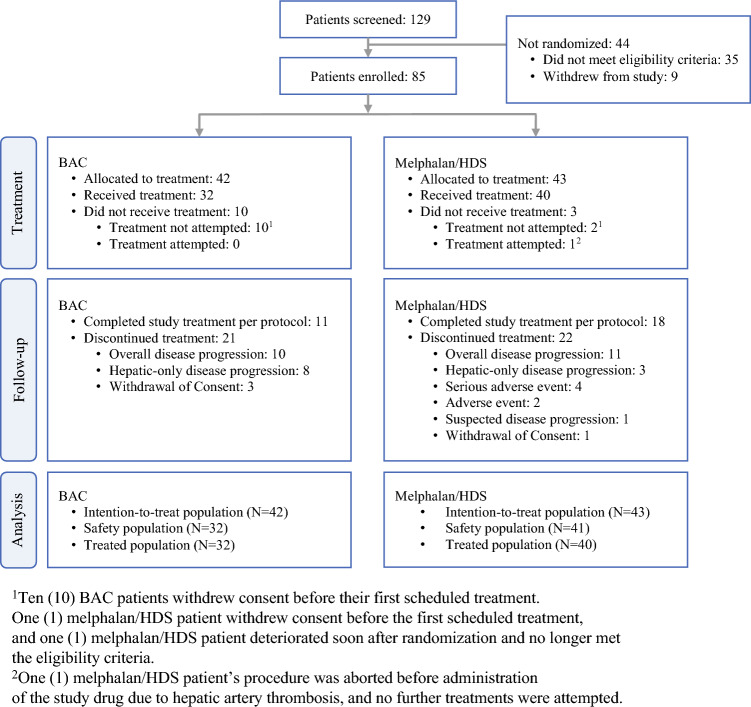
CONSORT diagram. Abbreviations: BAC, best alternative care; HDS, Hepatic Delivery System

Of the 42 patients were randomized to the BAC arm, 32 received treatment. In the treated population, 45% of patients treated with Melphalan/HDS completed the maximum of six cycles permitted per protocol; the primary reasons for discontinuation were disease progression (35.0%) and AEs/SAEs (15.0%). Of the treated BAC patients, 34.4% completed treatment per protocol; the primary reasons for discontinuation were disease progression (56.3%) and withdrawal of consent (9.4%) (Fig. 1).

At the time of the data cut-off of December 2, 2022, median duration of follow-up was 56.1 months, and 6.9% of the treated patients were still being followed for survival. In the treated patient population, key baseline disease characteristics were comparable between the two arms of the study (Table 1). Median time since diagnosis of primary tumor was 42.8 months for the Melphalan/HDS group) and 42.5 months for the BAC group. Median time from primary diagnosis to liver metastasis was 36.5 months for the Melphalan/HDS group and 35.3 months for the BAC group. Median time since diagnosis of liver metastasis was 5.4 months for the Melphalan/HDS group and 2.5 months for the BAC group. Median baseline hepatic tumor burden was 60.0 mm in the Melphalan/HDS group and 52.5 mm in the BAC group. Baseline extent of liver involvement (≤25% vs. 26–50%) was 77.5% versus 22.5%, respectively, for the Melphalan/HDS group and 75.0% versus 25.0%, respectively, for the BAC group. The number of hepatic lesions for the Melphalan/HDS versus the BAC patients was one (7.5% vs. 3.1%), two (25.0% vs. 31.3%) or three or more (67.5% vs. 65.6%). 12.5% of Melphalan/HDS and 21.9% of BAC patients had extrahepatic disease. Baseline lactate dehydrogenase (LDH) levels were elevated in 38.5% of Melphalan/HDS patients and 51.6% of BAC patients. Baseline ECOG status for Melphalan/HDS versus BAC patients was zero (85.0% vs. 71.9%), one (10.0% vs. 25.0%), and not recorded (5.0% vs. 3.1%). Before study entry, 47.5% of Melphalan/HDS patients and 43.8% of BAC patients had received prior therapy for their metastatic disease.

**Table 1:** Patient demographics and baseline characteristics (treated population)

Characteristic	Melphalan/HDS (*n* = 40)	BAC (*n* = 32)
Median age (range)—years	63.0 (20–78)	61.0 (31–82)
Male sex—no. (%)	20 (50.0)	14 (43.8)
Ethnicity—no. (%)		
Hispanic or Latino	1 (2.5)	2 (6.3)
Non-Hispanic or Latino	38 (95.0)	28 (87.5)
No response	1 (2.5)	2 (6.3)
Race—no. (%)		
White	39 (97.5)	30 (93.8)
Other	0 (0.0)	0 (0.0)
No response	1 (2.5)	2 (6.3)
Median time since primary diagnosis (range)—months^1^	42.8 (0.7–107.2)	42.5 (7.3–134.0)
Median time from primary diagnosis to liver metastasis (range)—months	36.5 (0.0–105.1)	35.3 (0.9–120.7)
Median time since diagnosis of liver metastases (range)—months^1^	5.4 (0.5–40.4)	2.5 (0.3–26.0)
ECOG performance status score—no. (%)		
0	34 (85.0)	23 (71.9)
1	4 (10.0)	8 (25.0)
Not recorded	2 (5.0)	1 (3.1)
Elevated LDH—no./*N* (%)	15/39 (38.5)	16/31 (51.6)
Extent of liver involvement—no. (%)^2^		
≤25%	31 (77.5)	24 (75.0)
26−50%	9 (22.5)	8 (25.0)
Median baseline hepatic tumor burden (mm)^2,3^	60.0	52.5
Largest hepatic lesion—no. (%)^2,4^		
≤ 3 cm (Stage M1a)	16 (40.0)	14 (43.8)
3.1 to 8 cm (Stage M1b)	19 (47.5)	17 (53.1)
≥ 8.1 cm (Stage M1c)	5 (12.5)	1 (3.1)
Extrahepatic lesions—no. (%)^2^	5 (12.5)	7 (21.9)
Lesion location—no. (%)		
Lymph node	3 (7.5)	0 (0.0)
Lung	2 (5.0)	2 (6.3)
Bone	1 (2.5)	3 (9.4)
Other visceral^5^	1 (2.5)	3 (9.4)
Soft tissue	1 (2.5)	2 (6.3)
Brain	0 (0.0)	0 (0.0)
Prior therapies—no. (%)^6^	19 (47.5)	14 (43.8)
Radiation	6 (15.0)	2 (6.3)
Surgery^7^	6 (15.0)	4 (12.5)
Systemic	12 (30.0)	11 (34.4)
Immune checkpoint inhibitor	10 (25.0)	7 (21.9)
Chemotherapy	1 (2.5)	3 (9.4)
TACE	1 (2.5)	0 (0.0)
Immunoembolization	0 (0.0)	1 (3.1)
SIRT	0 (0.0)	1 (3.1)

ECOG, Eastern Cooperative Oncology Group; LDH, lactate dehydrogenase; TACE, transarterial chemoembolization; SIRT, selective internal radiation therapy

^1^Months from diagnosis of either primary tumor or liver metastases to randomization

^2^Assessed by the investigator

^3^Based on sum of hepatic target lesion diameters. One Melphalan/HDS patient, who had no hepatic lesions ≥ 10 mm at baseline, was excluded from calculation of the median.

^4^Tumor staging per American Joint Committee on Cancer (AJCC Cancer) Staging Manual, 7th edition

^5^Includes stomach, pancreas, ovaries, and abdominal viscera.

^6^Patients with multiple therapies of a given type are counted once for that type.

^7^Includes only therapeutic surgeries/procedures and excludes non-therapeutic prior surgeries/procedures, e.g., biopsy. Each surgery/procedure was retrospectively classified as therapeutic or non-therapeutic

### Efficacy

**Fig. 2:**
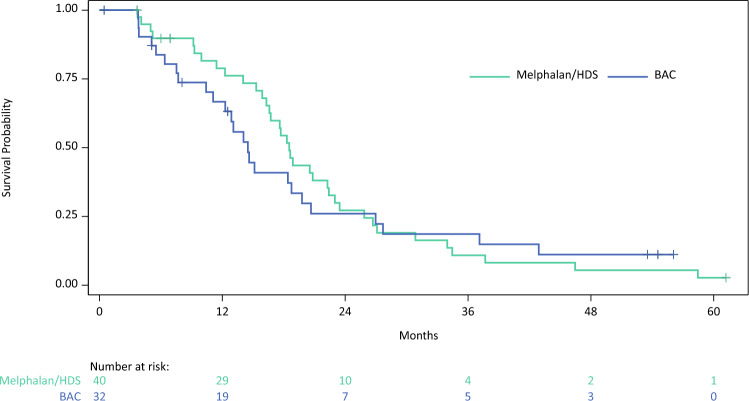
Kaplan–Meier plot of overall survival in patients treated with Melphalan/Hepatic Delivery System or BAC. Abbreviations: BAC, best alternative care; HDS, Hepatic Delivery System

**Fig. 3:**
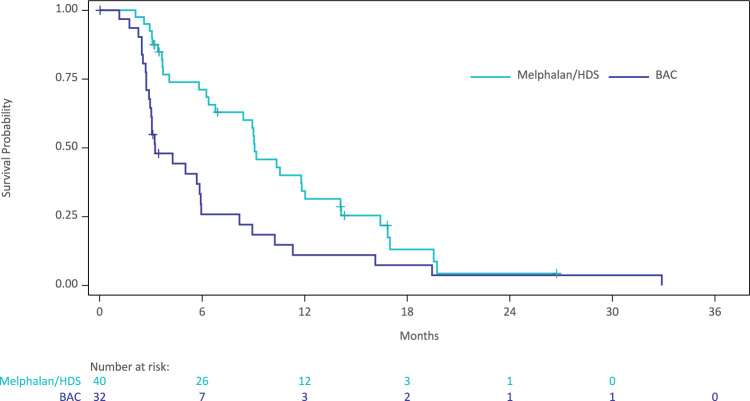
Kaplan–Meier plot of progression-free survival in patients treated with Melphalan/Hepatic Delivery System or BAC - Assessed by the investigator. Abbreviations: BAC, best alternative care; HDS, Hepatic Delivery System

All efficacy analyses (described in Table 2) were exploratory, as they were underpowered due to early termination of the randomized study. In the primary endpoint, mOS was numerically higher in patients treated with Melphalan/HDS (18.5 months) versus BAC (14.5 months) (Fig. 2). OS rate at 1 year numerically favored the Melphalan/HDS arm (79%) versus the BAC arm (67%). At 2 years, the OS rates were comparable (27% vs. 26%). mPFS was approximately three times longer in patients treated with Melphalan/HDS versus BAC (9.1 months vs. 3.3 months) (Fig. 3). The 6-month PFS rates were 71% versus 26%, and the 1-year PFS rates were 34% versus 11% in patients treated with Melphalan/HDS versus BAC, respectively.

**Table 2:** Clinical outcomes in patients treated with Melphalan/Hepatic Delivery System or BAC (Treated population—Assessed by the Investigator)

Characteristic	Melphalan/HDS (*n* = 40)	BAC (*n* = 32)
*Primary endpoint*		
Median overall survival—months [95% CI]^1^	18.5 [16.30, 22.41]	14.5 [11.10, 19.78]
*P*-value (Log-rank)	0.714	
Hazard ratio [95% CI]^2^	0.91 [0.54, 1.54]	
*P*-value (Chi-square)	0.728	
Overall survival at 1 year—% [95% CI]^1^	79 [62, 89]	67 [47, 81]
Overall survival at 2 years—% [95% CI]^1^	27 [14, 42]	26 [12, 43]
*Secondary endpoints*		
Median progression-free survival—months [95% CI]^1^	9.0 [6.37, 11.83]	3.1 [2.89, 5.91]
*P*-value (Log-rank)	0.015	
Hazard ratio [95% CI]^2^	0.35 [0.20, 0.61]	
*P*-value (Chi-square)	0.0002	
Progression-free survival at 6 months—% [95% CI]^1^	64 [54, 83]	32 [12, 43]
Progression-free survival at 1 year—% [95% CI]^1^	31 [20, 50]	11 [3, 26]
Objective response rate—% [95% CI]^3^	27.5 [14.60, 43.89]	9.4 [1.98, 25.02]
*P*-value (Fisher’s Exact)	0.074	
No. of patients who achieved objective response	11	3
Best overall response—no. (%)^4,5^		
Complete response	3 (7.5)	0 (0.0)
Partial response	8 (20.0)	3 (9.4)
Stable disease	21 (52.5)	12 (37.5)
Progressive disease	8 (20.0)	16 (50.0)
Not evaluable	0 (0.0)	1 (3.1)
*Exploratory endpoints*		
Median duration of response in responders—months [95% CI]^1^	14.0 [5.29, 14.29]; n = 11	5.6 [4.90, NC]; n = 3
*P*-value (log-rank)	0.003	
Hazard ratio [95% CI]^2^	0.07 [0.01, 0.81]	
*P*-value (Chi-square)	0.034	
Disease control rate—% [95% CI]^3^	80.0 [64.35, 90.95]	46.9 [29.09, 65.26]
*P*-value (Fisher’s Exact)	0.006	
No. of patients who achieved disease control	32	15
Median time to objective response—months [95% CI]^1^	3.1 [2.60, 6.21]; n = 11	8.4 [2.66, NC]; n = 3
Median hepatic progression-free survival—months [95% CI]^1^	11.4 [9.03, 15.90]	3.3 [2.89, 8.18]
*P*-value (log-rank)	0.0008	
Hazard ratio [95% CI]^2^	0.28 [0.16, 0.51]	
*P*-value (Chi-square)	<0.0001	
Hepatic objective response rate—% [95% CI]^3^	35.0 [20.63, 51.68]; n = 14	9.4 [1.98, 25.02]; n = 3

CI, confidence interval

^1^Kaplan-Meier estimate

^2^Hazard ratio is from a Cox regression model with main effect of treatment and with (US vs. Outside US) and extent of liver involvement (≤25% vs. 26–50%) as covariates. Hazard ratio is relative to BAC with <1 favoring Melphalan/HDS

^3^Patients without at least 1 post-baseline response assessment were designated as non-responders

^4^Best overall response (Response Evaluation Criteria in Solid Tumors v1.1) from the date of randomization until disease progression

^5^For complete response or partial response, confirmation was required by repeat assessment ≥4 weeks after initial documentation. To qualify as stable disease, the image must have been taken at least 9 weeks after start of therapy

Similarly, ORR and disease control rate (DCR) were numerically higher in the Melphalan/HDS arm versus the BAC arm, with ORRs of 27.5% versus 9.4% and DCRs of 80.0% versus 46.9%. mDOR was 14.0 months in the Melphalan/HDS arm and 5.6 months in the BAC arm.

### Safety

A total of 72 patients (*n* = 40 and *n* = 32, Melphalan/HDS and BAC, respectively) received study treatment, and 1 patient (Melphalan/HDS) had treatment attempted; therefore, 73 patients were assessed for safety (safety population; see safety results in Table 3). In the Melphalan/HDS arm, 14.6% of patients experienced treatment-emergent AEs (TEAEs) leading to discontinuation of study treatment, and 7.3% experienced TEAEs leading to dose reduction. No patients in the BAC arm experienced TEAEs leading to treatment discontinuation or dose reduction. Two patients died during the study, both in the Melphalan/HDS arm. The causes of death were acute hepatic failure and bacterial peritonitis, occurring at 62 and 64 days, respectively, after the last study treatment. Neither of the deaths were considered related to study treatment, device, or procedure.

All Melphalan/HDS patients and 93.8% of BAC patients experienced at least one TEAE; 85.4% of Melphalan/HDS and 34.4% of BAC patients had at least one severe (grade 3 or 4) TEAE. Hematological toxicity was the dominant toxicity in the Melphalan/HDS arm, with 56.1%, 36.6%, 36.6% and 34.1% of patients experiencing severe thrombocytopenia, leukopenia, neutropenia and anemia respectively. The only other severe TEAE occurring in ≥10% of patients in this arm was hypophosphatemia (14.6%). SAEs occurring in the Melphalan/HDS arm were also mostly hematological with 19.5%, 9.8%, 9.8% and 7.3% of patients experiencing serious thrombocytopenia, leukopenia, neutropenia and febrile neutropenia, respectively. Hematological toxicity was transient and manageable with standard supportive care.

**Table 3:** Treatment-emergent adverse events and serious adverse events in patients treated with Melphalan/Hepatic delivery system or BAC (safety population)

Parameters	Melphalan/HDS (*n* = 41)		BAC (*n* = 32)	
Any TEAE leading to discontinuation of study treatment	6 (14.6)		0 (0.0)	
Any TEAE leading to dose reduction of study treatment	3 (7.3)		0 (0.0)	
Death due to any AE	2 (4.9)		0 (0.0)	

	Any grade	Grade 3/4	Any grade	Grade 3/4
*Any TEAE*^1^	41 (100.0)	35 (85.4)	30 (93.8)	11 (34.4)
Nausea	29 (70.7)	0 (0.0)	19 (59.4)	1 (3.1)
Thrombocytopenia^2^	28 (68.3)	23 (56.1)	1 (3.1)	0 (0.0)
Anemia^3^	27 (65.9)	14 (34.1)	2 (6.3)	0 (0.0)
Fatigue	23 (56.1)	0 (0.0)	11 (34.4)	0 (0.0)
Vomiting	18 (43.9)	0 (0.0)	11 (34.4)	1 (3.1)
Leukopenia^4^	17 (41.5)	15 (36.6)	2 (6.3)	1 (3.1)
Neutropenia^5^	16 (39.0)	15 (36.6)	1 (3.1)	0 (0.0)
Abdominal pain upper	13 (31.7)	1 (2.4)	6 (18.8)	1 (3.1)
ALT increased	11 (26.8)	1 (2.4)	3 (9.4)	2 (6.3)
Back pain	11 (26.8)	0 (0.0)	3 (9.4)	0 (0.0)
INR increased	11 (26.8)	3 (7.3)	0 (0.0)	0 (0.0)
Diarrhea	9 (22.0)	0 (0.0)	1 (3.1)	0 (0.0)
Dyspnea	9 (22.0)	0 (0.0)	1 (3.1)	0 (0.0)
Contusion	8 (19.5)	0 (0.0)	0 (0.0)	0 (0.0)
Headache	8 (19.5)	0 (0.0)	4 (12.5)	0 (0.0)
Abdominal pain	7 (17.1)	0 (0.0)	8 (25.0)	1 (3.1)
Activated PTT prolonged	7 (17.1)	2 (4.9)	0 (0.0)	0 (0.0)
AST increased	7 (17.1)	1 (2.4)	3 (9.4)	2 (6.3)
Asthenia	7 (17.1)	0 (0.0)	4 (12.5)	0 (0.0)
Blood AP increased	7 (17.1)	1 (2.4)	4 (12.5)	0 (0.0)
Decreased appetite	7 (17.1)	0 (0.0)	4 (12.5)	0 (0.0)
Hypophosphatemia	7 (17.1)	6 (14.6)	0 (0.0)	0 (0.0)
Pain in extremity	7 (17.1)	0 (0.0)	1 (3.1)	0 (0.0)
Blood bilirubin increased	6 (14.6)	2 (4.9)	0 (0.0)	0 (0.0)
Hypocalcemia	6 (14.6)	2 (4.9)	0 (0.0)	0 (0.0)
Hypomagnesemia	6 (14.6)	0 (0.0)	0 (0.0)	0 (0.0)
Lethargy	6 (14.6)	0 (0.0)	0 (0.0)	0 (0.0)
Troponin I increased	6 (14.6)	1 (2.4)	1 (3.1)	0 (0.0)
Troponin T increased	6 (14.6)	3 (7.3)	0 (0.0)	0 (0.0)
Arthralgia	5 (12.2)	0 (0.0)	1 (3.1)	0 (0.0)
Dysgeusia	5 (12.2)	0 (0.0)	1 (3.1)	0 (0.0)
Hypoalbuminemia	5 (12.2)	1 (2.4)	1 (3.1)	0 (0.0)
Troponin increased	5 (12.2)	0 (0.0)	0 (0.0)	0 (0.0)
Electrocardiogram QT prolonged	3 (7.3)	3 (7.3)	0 (0.0)	0 (0.0)
Syncope	3 (7.3)	3 (7.3)	0 (0.0)	0 (0.0)
Pyrexia	4 (9.8)	0 (0.0)	5 (15.6)	0 (0.0)
Catheter site pain	3 (7.3)	0 (0.0)	4 (12.5)	0 (0.0)
Constipation	3 (7.3)	0 (0.0)	4 (12.5)	0 (0.0)
Hypertension	2 (4.9)	0 (0.0)	2 (6.3)	2 (6.3)
Anxiety	1 (2.4)	0 (0.0)	4 (12.5)	0 (0.0)
*Any serious TEAEs*^1^	21 (51.2)	17 (41.5)	7 (21.9)	5 (15.6)
Thrombocytopenia^2^	8 (19.5)	8 (19.5)	0 (0.0)	0 (0.0)
Leukopenia^4^	4 (9.8)	4 (9.8)	0 (0.0)	0 (0.0)
Neutropenia^5^	4 (9.8)	4 (9.8)	0 (0.0)	0 (0.0)
Febrile neutropenia	3 (7.3)	2 (4.9)	0 (0.0)	0 (0.0)
Cholecystitis	0 (0.0)	0 (0.0)	2 (6.3)	0 (0.0)
Nausea	0 (0.0)	0 (0.0)	2 (6.3)	0 (0.0)
Vomiting	0 (0.0)	0 (0.0)	2 (6.3)	0 (0.0)

TEAEs denote treatment-emergent adverse events, which are presented by maximum severity. Data presented as n (%) unless otherwise indicated.

ALT, alanine aminotransferase; AP, alkaline phosphatase; AST, aspartate aminotransferase; INR, international normalized ratio; PTT, partial thromboplastin time; TEAEs, treatment-related adverse events.

^1^TEAEs that were reported in at least 10% of patients (any grade) or in at least 5% of patients (Grade 3/4 and serious TEAEs) in either treatment arm.

^2^Thrombocytopenia includes thrombocytopenia, platelet count decreased.

^3^Anemia includes anemia, febrile bone marrow aplasia, hemoglobin decreased, normochromic normocytic anemia, red blood cell count decreased.

^4^Leukopenia includes leukopenia, lymphocyte count decreased, lymphopenia, white blood cell count decreased.

^5^Neutropenia includes neutropenia, neutrophil count decreased.

In the BAC arm, severe TEAEs included alanine aminotransferase (ALT) and aspartate aminotransferase (AST) elevation and hypertension (6.3% each) as well as nausea, vomiting, leukopenia, abdominal pain, and upper abdominal pain (3.1% each); and SAEs included cholecystitis, nausea and vomiting (6.3% each). None of the BAC-treated patients experienced hematological SAEs.

## Discussion

Metastatic uveal melanoma presents a therapeutic challenge. Among the limited treatment options is tebentafusp, a bispecific immunotherapeutic agent indicated for HLA-A*02:01-positive adult patients with unresectable mUM.^27^ Approximately 45% of mUM patients are HLA-A*02:01-positive, and for these patients tebentafusp is a treatment option. The only FDA-approved liver-directed treatment for patients with mUM is HEPZATO KIT, recently approved by the FDA based on the results from the FOCUS study. Because of its viability in mUM patients regardless of tumor genotype, HEPZATO KIT offers broad utility in the treatment of mUM. The FOCUS study evaluated PHP using the drug/device combination of Melphalan/HDS for treatment of patients with unresectable mUM. The PHP procedure isolates hepatic blood flow, delivers high-dose melphalan directly to the liver, and filters out residual melphalan, reducing systemic toxicity and limiting melphalan-related AEs.

The FOCUS study population was heterogenous and included patients who had hepatic disease with up to 50% of liver tumor involvement who also could have had limited extrahepatic disease. Demographic and baseline characteristics were generally similar between treatment arms, except for a numerical difference in the percentage of patients with extrahepatic disease at baseline (Melphalan/HDS arm: 12.5%; BAC arm: 21.9%). In the Melphalan/HDS arm, there was a nearly even split between patients who were previously treated (47.5%) and those who were treatment-naïve (52.5%) (Table 1).

The diverse study population, along with operational conduct at 22 study centers was expected to provide a robust evaluation of efficacy and safety of Melphalan/HDS in patients with unresectable mUM. Slow recruitment and reluctance of patients to receive BAC treatment led to early termination of the randomized study. Thus, all efficacy analyses presented in this report are exploratory and, as such, are intended to provide directional information for future studies.

Efficacy endpoints (see Table 2), including mOS, 1-year OS rate, mPFS, 6-month and 1-year PFS rates, mDOR, ORR and DCR were consistently numerically higher in the Melphalan/HDS arm than in the BAC arm. After completion of study treatment, data on subsequent therapies patients may have received were not collected, so the potential effect of post-study therapies on OS is unknown. All other efficacy endpoints were based on imaging conducted prior to initiation of subsequent therapies.

Observed response rates in both arms of the FOCUS study are lower than those previously reported elsewhere. Analysis ORR results in the BAC arm showed that in the 25/32 BAC patients treated with TACE, ORR was 12%. While interpretation of post-hoc analyses of small subgroups and cross-trial comparisons are methodologically challenging, it is interesting to note that in a recent review of regional therapies for mUM, ORRs from 5 prospective clinical studies of TACE varied widely: 16%, 21%, 57%, 60% and 100%.^28^ Similarly, ORR of 27.5% in the Melphalan/HDS arm was lower than the ORRs of around 60% reported in several small clinical studies investigating the safety and efficacy of the PHP procedure using Melphalan/HDS.^19–23^ One factor that may have contributed to this difference is the large number of clinical sites in the FOCUS study that did not have prior experience with the PHP procedure, whereas prior studies were conducted in centers highly experienced with the PHP procedure. Furthermore, patient selection and methodological differences in study design and conduct might have also contributed to the observed differences in ORR.

Comparisons with results from other clinical studies evaluating liver-directed therapies in mUM patients are difficult given markedly different patient populations, e.g. exclusion of patients with extrahepatic disease and other methodological differences. The EORTC 18021 study, in treatment-naïve patients, compared efficacy and safety of fotemustine administered intravenously or via hepatic intra-arterial (HIA) infusion. In the HIA arm, mOS was 14.6 months, mPFS was 4.5 months, ORR was 10.5%.^29^ In a double-blind, randomized Phase 2 study, the immunoembolization arm had a mOS of 21.5 months, a mPFS of 10.4 months, and an ORR of 21.2%.^30^

Review of safety results showed that patients treated with Melphalan/HDS generally had more AEs and more severe AEs than patients treated with BAC. Most of the AEs were hematological. While blood filtration after liver perfusion removes 86%^31^ of administered melphalan dose (up to 220 mg per treatment), the residual melphalan is the likely reason that a significant proportion of patients experienced severe myelosuppression; the observed safety profile is consistent with previous experience at these exposure levels.^32,33^ The hematological AEs were generally transient in nature, with nadirs at the end of the second week after treatment, and were mostly treated as an outpatient with observation.

Tolerability of Melphalan/HDS is good, as evidenced by a median of 4.5 completed treatment cycles and 45% of the patients completing the planned 6 treatment cycles as per study protocol. No new safety signals for Melphalan/HDS were reported.

An area of ongoing investigation is the combination of Melphalan/HDS treatment with ICI (ipilimumab and nivolumab) in mUM patients. Tong and colleagues recently published results from a small Phase 1b clinical study evaluating the combination of Melphalan/HDS with ipilimumab and nivolumab, indicating a good safety profile for this combination, as well as an intriguing early activity signal (DCR, 100%; ORR, 86%).^34^ The rationale for combining chemotherapy with ICI is based on the ability of Melphalan/HDS to enhance antigen presentation from killed cancer cells. This may result in immunomodulation, whereas anti–CTLA-4 and anti PD-1 antibodies enhance immune responses to released and circulating tumor antigens with activation of tumor-reactive immune cells.

Melphalan/HDS is a promising liver-directed treatment option for unresectable mUM. Additional clinical studies in other tumor types with unresectable hepatic metastases and combinations with immunotherapy are needed to further explore the full clinical potential of this novel treatment approach.

## Conclusions

The randomized FOCUS study provides evidence of the potential for clinical benefit of Melphalan/HDS in patients with unresectable mUM. This therapy offers a treatment option for patients with this rare condition which is associated with a poor prognosis and limited treatment options. Overall, the results demonstrate an acceptable benefit-risk profile for Melphalan/HDS in this patient population. A formal statistical analysis was not feasible due to early termination of the study and the small sample (85 enrolled vs. 240 planned).

## Supplementary Information

Below is the link to the electronic supplementary material.

Supplementary file1 (DOCX 48 KB)

## Data Availability

The data that support the findings of this study are available from the corresponding author, JSZ, upon reasonable request.
