# Supplementary material for: An Open-label, Randomized Study of Melphalan/Hepatic Delivery System Versus Best Alternative Care in Patients with Unresectable Metastatic Uveal Melanoma
Source: Ann Surg Oncol. 2025 Apr 7;32(7):4976–88. doi: 10.1245/s10434-025-17231-x (PMC12130151; doi:10.1245/s10434-025-17231-x)
Supplement: Supplementary file 1 — Supplementary file1 (DOCX 48 KB) [file 10434_2025_17231_MOESM1_ESM.docx]

# An Open-label, Randomized Study of Melphalan/Hepatic Delivery System Versus Best Alternative Care in Patients with Unresectable Metastatic Uveal Melanoma

Jonathan S. Zager^1,2*^, Marlana Orloff^3^, Pier Francesco Ferrucci^4^, Junsung Choi^2,5^, David J. Eschelman^3^, Evan S. Glazer^6^, Aslam Ejaz^7^, J. Harrison Howard^8^, Erika Richtig^9^, Sebastian Ochsenreither^10^, Sunil A. Reddy^11^, Michael C. Lowe^12^, Georgia M. Beasley^13^, Anja Gesierich^14^, Armin Bender^15^, Martin Gschnell^15^, Reinhard Dummer^16^, Michel Rivoire^17^, Ana Arance^18^, Stephen William Fenwick^19^, Joseph J. Sacco^20^, Sebastian Haferkamp^21^, Carsten Weishaupt^22^, Johnny John^23^, Matthew Wheater^24^, Christian H. Ottensmeier^20^

^1^Department of Cutaneous Oncology, Moffitt Cancer Center, Tampa, FL, USA

^2^Department of Oncologic Sciences, University of South Florida, Morsani College of Medicine, Tampa, FL, USA

^3^Thomas Jefferson University, Philadelphia, PA, USA

^4^European Institute of Oncology, IRCCS, Milan, Italy

^5^Moffitt Cancer Center, Tampa, FL, USA

^6^The University of Tennessee Health Science Center, Memphis, TN, USA

^7^The Ohio State University, Columbus, OH, USA

^8^University of South Alabama, Mobile, AL, USA

^9^Medical University of Graz, Graz, Austria

^10^Charité Comprehensive Cancer Center, Berlin, Germany

^11^Stanford University, Stanford, CA, USA

^12^Emory University, Atlanta, GA, USA

^13^Duke University, Durham, NC, USA

^14^University Hospital Würzburg, Würzburg, Germany

^15^University Hospital Marburg, Marburg, Germany

^16^University Hospital Zürich, Zürich, Switzerland

^17^Léon Bérard Center, Lyon, France

^18^Hospital Clínic Barcelona, Barcelona, Spain

^19^Liverpool University Hospitals, NHS Foundation Trust, Liverpool, UK

^20^The Clatterbridge Cancer Center, University of Liverpool, Liverpool, UK

^21^University Hospital Regensburg, Regensburg, Germany

^22^University Hospital Münster, Münster, Germany

^23^Delcath Systems, Inc., Queensbury, USA

^24^University Hospital Southampton, NHS Foundation Trust, Southampton, UK

SUPPLEMENTARY APPENDIX

1. Inclusion/exclusion criteria
2. Study treatment: melphalan/Hepatic Delivery System (melphalan/HDS)
3. Dose and schedule of melphalan/HDS treatment
4. Best alternative care (BAC) treatment assignments (treated population)
5. References

# **Inclusion/exclusion criteria**

## Inclusion criteria

Subjects were to meet all of the following criteria for study entry:

- Male or female subjects ≥18 years of age.
- Subjects must weigh ≥35 kg (due to possible size limitations with respect to percutaneous catheterization of the femoral artery and vein using the Delcath Hepatic Delivery System [HDS]).
- 50% or less histologically or cytologically proven ocular melanoma (OM) metastases in the parenchyma of the liver.
- Disease in the liver measurable by computed tomography (CT) scan and/or magnetic resonance imaging (MRI).
- Evidence of limited extrahepatic disease on preoperative radiological studies was acceptable if the life-threatening component of disease was in the liver. Limited extrahepatic disease was defined as follows: metastasis in bone, subcutaneous, lung, or lymph nodes that was amenable to resection or radiation and had a defined treatment plan.
- Scans used to determine eligibility (CT scan of the chest/abdomen/pelvis and MRI of the liver) performed within 28 days prior to eligibility. An MRI of the liver was required at Screening to validate that CT scan accurately reflects the extent of disease in the liver. For subjects with MRI intolerance, a 3-phase liver CT scan was to be done in place of liver MRI.
- Subjects with no chemotherapy, radiotherapy, chemoembolization, radioembolization, or immunoembolization for their malignancy within 30 days prior to treatment and recovered from all side effects of therapeutic and diagnostic interventions except those listed in Appendix B of the study protocol.
- Subjects receiving anti-programmed cell death protein 1 immunotherapy, such as pembrolizumab or nivolumab, or human cytotoxic T-lymphocyte antigen 4 blocking antibody, such as ipilimumab, were to have completed treatment 8 weeks prior to study enrolment.
- Subjects with an Eastern Cooperative Oncology Group Performance Status (ECOG PS) score of 0 to 1 at Screening.
- Subjects with adequate hepatic function as evidenced by all of the following: total serum bilirubin ≤1.5× the upper limit of normal (ULN) and a prothrombin time within 2 seconds above the ULN and aspartate aminotransferase (AST)/alanine aminotransferase (ALT) must be ≤2.5× ULN.
- Subjects must have all of the following: platelet count >100000/μL, hemoglobin ≥10.0 g/dL, white blood cell (WBC) count >2000/μL, absolute neutrophil count (ANC) ≥1.5×10^9^/L, and serum creatinine ≤1.5 mg/dL unless the measured creatinine clearance was >40 mL/min/1.73 m^2^.
- Women of childbearing potential with a negative serum pregnancy test (β-human chorionic gonadotropin) within 7 days prior to eligibility.
- Provided signed informed consent.

## Exclusion criteria

Subjects who met any of following criteria were to be excluded from study entry:

- Subjects with Child-Pugh Class B or C cirrhosis or with evidence of portal hypertension by history, endoscopy, or radiologic studies.
- Subjects who were unable to undergo general anesthesia for any reason. Those with New York Heart Association active cardiac conditions. An evaluation of risk was to occur for those with functional classification II, III, or IV. This included, but was not limited to, the following conditions: unstable coronary syndrome (unstable or severe angina or myocardial infarction within 6 months prior to Screening), worsening or new-onset congestive heart failure, significant arrhythmias, or severe valvular disease.
- History or evidence of clinically significant pulmonary disease that precluded the use of general anesthesia.
- Women of childbearing potential, i.e., fertile meaning not permanently sterilized and having had a menstrual period within the past 12 months, unable to undergo hormonal suppression to avoid menstruation during treatment.
- Women of childbearing potential and fertile males (not permanently sterile by bilateral orchidectomy) unwilling or unable to use highly effective contraception method for consent to at least 6 months after the last administration of study treatment (e.g., combined hormonal contraception, progestogen-only hormonal contraception, intrauterine device, intrauterine hormone-releasing system, bilateral tubal occlusion, vasectomized partner, or sexual abstinence).
- Females who were pregnant or breastfeeding.
- Subjects taking immunosuppressive drugs; however, oral corticosteroids ≤10 mg/day were allowed.
- Subjects unable to be temporarily removed from chronic anticoagulation therapy.
- Subjects with active bacterial infections with systemic manifestations (malaise, fever, or leukocytosis) were not eligible until completion of appropriate therapy.
- Subjects with severe allergic reaction to iodine contrast, which cannot be controlled by premedication with antihistamines and steroids.
- Subjects with a history of or known hypersensitivity to melphalan or the components of melphalan/HDS.
- Subjects with a latex allergy.
- Subjects with a history of hypersensitivity to heparin or the presence of heparin-induced thrombocytopenia.
- Subjects with a history of bleeding disorders or evidence of intracranial abnormalities that would put them at risk for bleeding with anticoagulation (e.g., strokes or active metastases).
- Subjects with a history of gastrinoma. Note: For subjects with a history of liver surgery or major vasculature surgery, a CT scan or MR angiogram was required during Screening to assure that the subject did not have hepatic vasculature incompatible with perfusion, hepatofungal flow in the portal vein, or known unresolved venous shunting.
- Known varices at risk of bleeding, including medium or large esophageal or gastric varices or active peptic ulcer.
- Subjects with prior Whipple’s procedure.
- Subjects with brain metastases or presence of other intracranial lesions at risk for bleeding by history or baseline radiologic imaging.
- Subjects with an active liver infection, including hepatitis B and hepatitis C infection.
- Subjects with anti-hepatitis B core antibody positive or hepatitis B surface antigen but DNA negative were exception(s).
- Uncontrolled endocrine disorders including diabetes mellitus, hypothyroidism, or hyperthyroidism.
- Received any investigational agent for any indication within 30 days prior to the first treatment.
- Not recovered from side effects of prior therapy to ≤Grade 1 (according to National Cancer Institute [NCI] Common Terminology Criteria for Adverse Events [CTCAE] v.4.03). Certain side effects that were unlikely to develop into serious or life-threatening events (e.g., alopecia) are allowed at >Grade 1.
- Cancers other than OM for which the subject was currently under treatment or still deemed to be not cancer-free.

# Study treatment: melphalan/Hepatic Delivery System (melphalan/HDS)

The melphalan/HDS is a co-packaged drug-device combination product. Melphalan is a bifunctional alkylating agent with broad efficacy as an anticancer chemotherapeutic agent against various tumor histologies. It is well-studied and has limited liver toxicity, a high hepatic extraction rate, a short half-life, and an immediate apoptosis effect on tumor cells.^1,2^ The HDS consists of an extracorporeal hemofiltration circuit (EFC), an infusion catheter to deliver melphalan to the hepatic artery, and a femoral access set. The EFC is a closed circuit of catheters that lowers the melphalan concentration in the hepatic venous blood before it is returned to the systemic circulation. The EFC includes a double-balloon catheter placed in the retro-hepatic inferior vena cava to isolate the hepatic venous blood from the systemic circulation, a hemofiltration circuit including hemofiltration cartridges to adsorb melphalan and a venous return line. This procedure of separating the liver with the double-balloon catheter, perfusion with melphalan, and hepatic venous blood filtration is called percutaneous hepatic perfusion.^3^

# Dose and schedule of melphalan/HDS treatment

Patients with unresectable hepatic-dominant metastatic uveal melanoma (UM) received melphalan (3.0 mg/kg ideal body weight; maximum dose: 220 mg for a single treatment) once every 6-8 weeks for a maximum of 6 cycles, with an acceptable delay of 2 weeks (i.e., 8 weeks in total) between cycles to allow for recovery from melphalan-related toxicity. Melphalan dose was calculated as shown in the table below.

***Ideal body weight calculation***

| **Gender** | **Height** | **Ideal body weight** |
| --- | --- | --- |
| Male | ≥152 cm | 52 kg + 0.75 kg/cm of height >152 cm |
|  | <152 cm | 52 kg – 0.75 kg/cm of height <152 cm |
| Female | ≥152 cm | 49 kg + 0.67 kg/cm of height >152 cm |
|  | <152 cm | 49 kg – 0.67 kg/cm of height <152 cm |

Each treatment cycle consisted of 6 weeks with a window of ±2 weeks. The second to sixth treatments were not administered if one of the following criteria applied:

- Hepatic disease progression
- Extrahepatic disease progression
- Treatment delay of 2 weeks (i.e., 8 weeks after the last melphalan/HDS treatment) due to prolonged toxicity

Tumor response was assessed every 12 weeks (±2 weeks) until disease progression. Imaging modalities used in Screening assessments were used to assess tumor response during the treatment period and in follow up. Patients with hepatic or extrahepatic progressive disease were discontinued from study treatment, and all patients were followed until death. The maximum duration of the study treatment phase for any patient was 12 months. Once a patient finished their treatments, they entered the follow-up period. If the patient had not progressed after their last treatment cycle, disease assessments were continued every 12 weeks (±2 weeks) until disease progression was documented. Meanwhile, if a patient had progressed after their last treatment cycle, their follow-up for survival status was done either in person or by phone every 3 months until death.

The dose of melphalan was to be reduced to 2.0 mg/kg for subsequent cycles if any of the following toxicities was observed:

- Grade 4 neutropenia lasting >5 days despite granulocyte growth factor support or Grade 3 or higher febrile neutropenia with granulocyte growth factor support.
- Grade 4 thrombocytopenia lasting >5 days or Grade 3 thrombocytopenia associated with medically significant bleeding.

# Best alternative care (BAC) treatment assignments (treated population)

|  | BAC (*N*=32) |
| --- | --- |
| Treatment - n (%) |  |
| TACE | 25 (78.1) |
| Pembrolizumab | 6 (18.8) |
| Ipilimumab | 1 (3.1) |
| Dacarbazine | 0 (0.0) |
| BAC, best alternative care; TACE, transarterial chemoembolization. | |

# References

1. Shah GL, Boelens JJ, Carlow D, Lin A, Schofield R, Sitner NC, et al, Population pharmacokinetics of melphalan in a large cohort of autologous and allogeneic hematopoietic cell transplantation recipients: towards individualized dosing regimens. *Clin Pharmacokinet* (2022) 61(4):553–63. doi: 10.1007/s40262-021-01093-z
2. ALKERAN^®^ (melphalan hydrochloride) for Injection [package insert]. GlaxoSmithKline 2011. U.S. Food and Drug Administration website. Available at <https://www.accessdata.fda.gov/drugsatfda_docs/label/2011/020207s016lbl.pdf> (Accessed May 21, 2024).
3. Modi S, Gibson T, Vigneswaran G, Patel S, Wheater M, Karydis I, et al. Chemosaturation with percutaneous hepatic perfusion of melphalan for metastatic uveal melanoma. *Melanoma Res* (2022) 32(2):103–11. doi: 10.1097/CMR.0000000000000806
4. DerSimonian R, Laird N. Meta-analysis in clinical trials. *Control Clin Trials* (1986) 7(3):177–88. doi: 10.1016/0197-2456(86)90046-2
5. Borenstein M, Hedges LV, Higgins JPT, et al: Introduction to Meta-analysis (Chapter 16). Chichester, West Sussex, U.K.; Hoboken: John Wiley & Sons; 2009.
6. Luke JJ, Callahan MK, Postowet MA, Romano E, Ramaiya N, Bluth M, et al. Clinical activity of ipilimumab for metastatic uveal melanoma: a retrospective review of the Dana-Farber Cancer Institute, Massachusetts General Hospital, Memorial Sloan-Kettering Cancer Center and University Hospital of Lausanne experience. *Cancer* (2013) 119(20):3687–95. doi: 10.1002/cncr.28282
7. Maio M, Danielli R, Chiarion-Sileni V, Pigozzo J, Parmiani G, Ridolfi R, et al. Efficacy and safety of ipilimumab in patients with pre-treated, uveal melanoma. *Ann Oncol* (2013) 24(11):2911–15. doi: 10.1093/annonc/mdt376
8. Zimmer L, Vaubel J, Mohr P, Hauschild A, Utikal J, Simon J, et al. Phase II DeCOG-study of ipilimumab in pretreated and treatment-naïve patients with metastatic uveal melanoma. *PLoS One* (2015) 10(3):e0118564. doi: 10.1371/journal.pone.0118564
9. Danielli R, Ridolfi R, Chiarion-Sileni V, Queirolo P, Testori A, Plummer R, et al. Ipilimumab in pretreated patients with metastatic uveal melanoma: safety and clinical efficacy. *Cancer Immunol Immunother* (2012) 61(1):41–8. doi: 10.1007/s00262-011-1089-0
10. Wiater K, Switaj T, Mackiewicz J, Kalinka-Warzocha E, Wojtukiewicz M, Szambora P, et al. Efficacy and safety of ipilimumab therapy in patients with metastatic melanoma: a retrospective multicenter analysis. *Contemp Oncol (Pozn)* (2013) 17(3):257–62. doi: 10.5114/wo.2013.35785
11. Khattak MA, Fisher R, Hughes P, Gore M, Larkin J. Ipilimumab activity in advanced uveal melanoma. *Melanoma Res* (2013) 23(1):79–81. doi: 10.1097/CMR.0b013e32835b554f
12. Rozeman EA, Fanchi L, van Akkooi ACJ, Kvistborg P, Thienen JV, Stegenga B, et al. (Neo) adjuvant ipilimumab + nivolumab (IPI+NIVO) in palpable stage 3 melanoma – updated relapse free survival (RFS) data from the OpACIN trial and first biomarker analysis. *Ann Oncol* (2017) 28 (suppl_5):v428–48.
13. Karydis I, Chan PY, Wheater M, Arriola E, Szlosarek PW, Ottensmeier CH. Clinical activity and safety of pembrolizumab in ipilimumab pre-treated patients with uveal melanoma. *Oncoimmunology* (2016) 5(5):e1143997. doi: 10.1080/2162402X.2016.1143997
14. Kottschade LA, McWilliams RR, Markovic SN, Block MS, Bisneto JV, Pham AQ, et al. The use of pembrolizumab for the treatment of metastatic uveal melanoma. *Melanoma Res* (2016) 26(3):300–3. doi: 10.1097/CMR.0000000000000242
15. Algazi AP, Tsai KK, Shoushtari AN, Munhoz RR, Eroglu Z, Piulats JM, et al. Clinical outcomes in metastatic uveal melanoma treated with PD-1 and PD-L1 antibodies. *Cancer* (2016) 122(21):3344–53. doi: 10.1002/cncr.30258
16. Heppt MV, Steeb T, Schlager JG, Rosumeck S, Dressler C, Ruzicka T, et al. Immune checkpoint blockade for unresectable or metastatic uveal melanoma: a systematic review. *Cancer Treat Rev* (2017) 60:44–52. doi: 10.1016/j.ctrv.2017.08.009
17. van der Kooij MK, Joosse A, Speetjens FM, Hospers GAP, Bisschop C, de Groot JWB, et al. Anti PD1 treatment in metastatic uveal melanoma in the Netherlands. *Acta Oncol* (2017) 56(1):101–3. doi: 10.1080/0284186X.2016.1260773
18. Bender C, Enk A, Gutzmer R, and Hassel JC. Anti-PD-1 antibodies in metastatic uveal melanoma: a treatment option? *Cancer Med* (2017) 6(7):1581–86. doi: 10.1002/cam4.887
19. González-Cao M, Arance A, Piulats JM, Marquez-Rodas, Manzano JL, Berrocal A, et al. Spanish Melanoma Group. Pembrolizumab for advanced melanoma: experience from the Spanish Expanded Access Program. *Clin Transl Oncol* (2017) 19(6):761–68. doi: 10.1007/s12094-016-1602-1
20. Piperno-Neumann S, Diallo A, Etienne-Grimaldi MC, Bidard F-C, Rodrigues M, Plancher C, et al. Phase II trial of bevacizumab in combination with temozolomide as first-line treatment in patients with metastatic uveal melanoma. *Oncologist* (2016) 21(3):281–2. doi: 10.1634/theoncologist.2015-0501
21. KEYTRUDA (pembrolizumab) Summary of Product Characteristics. Merck Sharp & Dohme B.V. Available at [Keytruda, INN-pembrolizumab (europa.eu)](https://www.ema.europa.eu/en/documents/product-information/keytruda-epar-product-information_en.pdf)
